# Examining differentials in HIV transmission risk behaviour and its associated factors among men in Southern African countries

**DOI:** 10.1057/s41599-022-01312-3

**Published:** 2022-08-27

**Authors:** Million Phiri, Musonda Lemba, Chrispin Chomba, Vincent Kanyamuna

**Affiliations:** 1 Department of Population Studies, School of Humanities and Social Sciences, University of Zambia, Lusaka, Zambia; 2 Department of Demography and Population Studies, Schools of Public Health and Social Sciences, University of the Witwatersrand, Johannesburg, South Africa; 3 Department of Community and Family Medicine, School of Public Health, University of Zambia, Lusaka, Zambia; 4 Department of Development Studies, School of Humanities and Social Sciences, University of Zambia, Lusaka, Zambia

**Keywords:** Social science, Sociology, Social policy

## Abstract

Sub-Saharan Africa (SSA), particularly Southern and East Africa, has the highest AIDS deaths and HIV-infected people in the world. Even though considerable effort has been made over the years to study HIV transmission risk behaviours of different population groups in SSA, there is little evidence of studies that have looked at pooled effects of associated HIV risk factors among men, particularly in Southern Africa. Thus, this study sought to fill this gap in knowledge by investigating the variations in HIV risk behaviours among men in the region. The study analysed cross-sectional data based on the most recent country Demographic and Health Survey (DHS) for six countries, namely Lesotho, Mozambique, Namibia, South Africa, Zambia and Zimbabwe. The study employed multivariate logistic regression models on a pooled dataset and individual country data to examine the relative risk of education and other factors on HIV risk behaviour indicators. It considered: (i) condom use during high risk-sex, (ii) multiple sexual partnerships, and (iii) HIV testing among men aged 15–59 years. Findings show that the proportion of men who engaged in HIV transmission risk behaviour was high in Southern Africa. Two-thirds of men reported non-use of a condom during last sex with most recent partners while 22% engaged in multiple sexual partnerships. The percentage of men who used condoms during sex with most recent partners ranged from 18% in Mozambique to 58% in Namibia. Age, residence, marital status and household wealth status were associated with HIV risk factors in the region. The study has established country variations in terms of how individual factors influence HIV transmission risk behaviour among men. Results show that the level of education was associated with increased use of condoms, only in Zambia and Mozambique. Delay in starting a sexual debut was associated with reduced odds of having multiple sexual partnerships in the region. Suggesting the need to strengthen comprehensive sexuality education among young men in school, to promote social behaviour change during adolescence age. The study presents important results to inform direct health policy, programme and government action to address HIV prevalence in the Southern region of Africa.

## Background

As the world commemorates the Fortieth World AIDS Day, the Human Immunodeficiency Virus (HIV) statistics are still staggering around the globe. Since its discovery in the 1980s, HIV and acquired immune deficiency syndrome (AIDS) has globally claimed about 32 million people (UNAIDS, 2020a, 2013aa; WHO, 2015). The Joint United Nations Programme on HIV/AIDS (UNAIDS) estimates show that about 37.9 million people were living with HIV in 2019, and of these, 28.5 million were in sub-Saharan Africa (UNAIDS, 2020a). UNAIDS estimates that over 70% of the global total of HIV-positive people live in sub-Saharan Africa (SAfAIDS, 2015; UNAIDS GH, 2019). HIV prevalence for males aged 15–59 years in Southern Africa is about 14% (UNAIDS, 2013ab). Even though the pandemic’s severe health effects have been managed by the advent of antiretroviral treatment (ART), many countries in Eastern and Southern Africa have not yet reached the HIV epidemic control level (UNAIDS, 2020b, 2013ab; WHO, 2015).

Epidemiological studies on HIV/AIDS published early in the late 1980s in sub-Saharan Africa reported formal education as a risk factor: educated sub-Saharan Africans had a higher chance of developing HIV/AIDS than their less educated counterparts (Baker et al., 2008; Leon et al., 2017). According to later demographic research, the effect of education on HIV transmission risk had reversed by the mid-1990s, later education functioned as a positive social factor helping men and women to have comprehensive knowledge of HIV prevention strategies (Baker et al., 2008; Gregson et al., 2007; Leon et al., 2017). Recent counter-evidence reveals a curvilinear pattern, with the link between educational attainment and HIV/AIDS infection risk, shifting from positive to negative as one progresses through the educational system (Gregson et al., 2001; Leon et al., 2017; Pettifor et al., 2008). However, this contracting finding in literature requires further repeated population level and longitudinal studies to validate the association between education level and other social independent correlates, conclusively.

Governments in the region, with support from the US President’s Emergency Plan for AIDS Relief (PEFFAR), United Nations Joint Programme on HIV/AIDS (UNAIDS), Global Fund and other international and local organisations have been implementing various HIV prevention programmes, targeted at ensuring that men have access to comprehensive information, encouraging abstinence from sex for the non-married, promote delay in initiation of sexual debut among school-going youths, encourage adults to stick to one equally faithful and uninfected sexual partner, and consistent use of condoms (SAfAIDS, 2015; Simona et al., 2022; UNAIDS, 2012, 2013ab; WHO, 2015) as well as the uptake of medical male circumcision (Garenne and Matthews, 2020; Phiri, 2011). Some studies have pointed out that promoting access to education is considered a priority intervention measure for reducing the risk of transmitting HIV among men. For instance, available research reveals a negative linear relationship between education attainment (years of education) and HIV infection rate (De Neve et al., 2015; Leon et al., 2017; Zuilkowski and Jukes, 2012). Despite Southern Africa having the highest HIV prevalence rates in the world, there is little research that has focused on understanding a comprehensive regional picture of the role of education and other social and behavioural factors in influencing HIV risk behaviour, especially in sexually active men. Considering the high prevalence of HIV rates among men in the Southern part of Africa, comparatively to other sub-regions in the continent and elsewhere (Awopegba et al., 2021; Johnson et al., 2017; Olakunde et al., 2020a), it is relevant that research aiming at investigating factors that predispose men to the risks of HIV infection should be of top priority in the region.

Although the prevalence of HIV in men appears to be falling over time globally, it remains unacceptably high in Southern and Eastern Africa (SAfAIDS, 2015; UNAIDS, 2020a; WHO, 2015). These levels of HIV prevalence in men, signify the fact that more effort in sexual and reproductive health programming at the health policy level needs to be done to stimulate a further reduction in HIV prevalence among men in Southern African (Baral and Phaswana-Mafuya, 2012; Hargreaves et al., 2008). Doing so requires an all-inclusive understanding of the determinants associated with HIV risk behaviour among men. Thus, the understanding of such factors affecting men is considerably lacking when we consider individual-level factors at the sub-regional level.

Therefore, this study was designed to fill the knowledge gap and come up with policy and programming suggestions aimed at further reducing HIV prevalence in men in Southern Africa. This study investigated factors associated with HIV transmission risk behaviour in men in the region. Furthermore, the study sought to establish if there were variations in HIV transmission risk behaviours across countries included in the analysis. Unlike similar studies conducted in SSA (Baker et al., 2008; Glynn et al., 2004; Leon et al., 2017; Lucas and Wilson, 2019), this study incorporates pooled comprehensive complex regional data to examine individual-level and contextual factors that influence HIV risk behaviours in men. The study achieved its objectives by making use of nationally representative cross-sectional surveys conducted in Southern African countries.

## Methods and data

### Data source

**Fig. 1:**
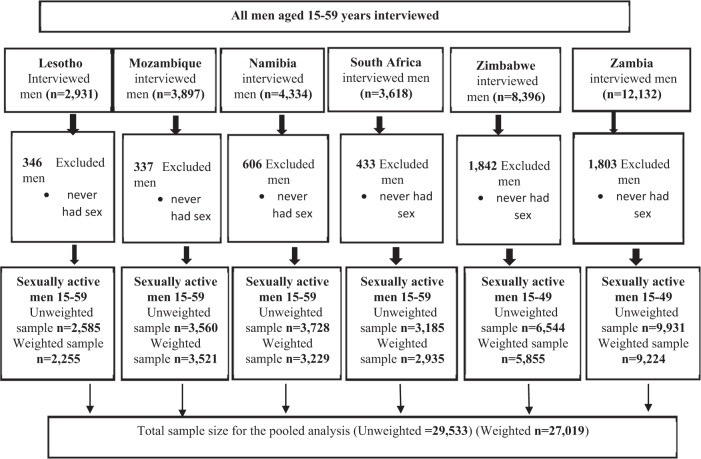
Sample derivation diagram.

This study used data extracted from the recent Demographic and Health Survey (DHS) datasets from six countries in Southern Africa, namely Lesotho, Mozambique, Namibia, South Africa, Zambia, and Zimbabwe (Fig. 1). The DHS programme is conducted in many developing countries and draws national representative samples of households that are usually selected via a two-stage stratified cluster sampling technique (Croft et al., 2018). Women aged 15–49 and men aged 15–59 years are usually selected for interviews in all sampled households. To facilitate comparison of indicators across countries, interviews are usually conducted using three standard questionnaires, namely; household questionnaire, woman questionnaire and men questionnaire. Participants in the DHS survey were interviewed by field workers, who were well-versed in a wide range of topics, including sexuality, HIV and AIDS knowledge and awareness, fertility preferences and family planning, and other reproductive health topics. DHS data are typically weighted to account for the complexity of survey design and response bias, with the goal of ensuring that the sample represents the general population (DHS programme, 2021). The total number of men aged 15–59 years included in this study is 29,533 (weighted = 27,019). Figure 1 shows the sampling derivation steps.

### Sample description

Analysis samples for this study comprised males aged 15–59 years extracted from each country’s recent DHS. Data came from the men’s recode files (MR Datasets) for each country. The sample included all men who were sexually active at the time of the survey. This resulted in a pooled sample of 27,019 men included in the analysis. Weighted samples of sexually active men ranged from 2255 men in Lesotho to 9224 in Zambia (Fig. 1).

### Study measures

#### Outcome variables

Three outcome variables of interest were used to measure HIV risk behaviour in men, these are condom use during sex with the most recent partner; multiple sexual partnerships and HIV testing in the last 12 months prior to the survey. Prior research has revealed that promoting condom use and understanding barriers to consistent condom use remain priorities, especially among sexually active men who have sex with multiple partners (Campbell et al., 2016). Further, fear of HIV testing has been identified to play an important role in HIV transmission in Africa (Asaolu et al., 2016a; Olakunde et al., 2020b). The DHS collected this information from all sexually active men aged 15–59 years who were interviewed in the survey. To facilitate analysis, the study classified the outcome variables as binary, with “0” showing non-occurrence of the outcome and “1” showing occurrence of the outcome of interest. We coded condom use as “0” representing non-use and “1” for using. The DHS collected a variable for multiple sexual partners as a numeric response. For the study analysis, we recoded this variable into binary to reflect “0” as having one sexual partner and “1” as having two or more sexual partners. HIV testing in the last 12 months was coded as “0” did not take an HIV test and “1” representing uptake on an HIV test.

#### Independent variables

Based on the review of existing literature on HIV and AIDS in SSA and elsewhere, the study identified correlates at individual and household levels that could be potentially associated with HIV transmission risk factors among men in the region. These variables were classified as socio-economic, demographic and contextual factors. The DHS reference materials and data collection forms were used to identify the independent variables of interest presented in this section. The following independent variables were included in the study analysis: age of a man categorised as (15–24, 25–34, 35–44, 45–54 and 55–59); current marital status (categorised as never married, currently married/living with a partner and formally married; place of residence (urban, rural); education level (no education, primary, secondary, tertiary); literacy (literate, illiterate); household wealth index, this variable is usually captured in the DHS using 5 response categories (poorest, poor, middle, rich, richest). For the analysis, we recoded this variable with the following categorisation (poor, middle, rich); employment status was categorised as (employed, unemployed); age at first sex was coded as (below 15 years, 15–24 years and 25+ years) and circumcision status (yes, no).

### Statistical analysis

Data analysis was performed using Stata SE version 17.0 statistical software. All analyses applied “svy” command to account for complex survey design, which considered sample weight. Descriptive analyses were presented using percentages and counts. Cross tabulations with *Chi*-square tests were conducted on country-specific data to explore the bivariate association between explanatory factors and exposure variables and establish the significance of the relationships across countries. Multivariate binary logistic regression to examine the determinants of HIV risk behaviours among men in the region. The choice of this model was informed by the dichotomous distribution of the dependent variables. Further, a regression model was fitted with all independent correlates identified in the literature. Multivariate logistic regression models were performed to examine the influence of education on HIV risk factors within countries. Based on the regression model, the odds ratios (OR) were calculated, along with their respective 95% confidence intervals (95% CI).

### Ethics

This study’s datasets were obtained from the DHS programme’s website (https://dhsprogram.com/), which is freely accessible to the public. Permission to use the dataset was obtained through the study’s registration with the DHS programme. The countries followed all ethical approvals before conducting their respective DHSs. The original DHS biomarker and survey protocols were approved by the respective countries’ Ethical Review Bodies and the Research Ethics Review Board of the Center for Disease Control and Prevention (CDC) Atlanta. There was no need for a separate ethical approval because the study used secondary datasets that did not contain any personally identifying information about the participants.

## Results

### Condom use during sex with most recent partner

Results show condom use during sex with the most recent partner varied across countries. The percentage ranged from 18.2% in Mozambique to 59.1% in Namibia. In almost all countries in Southern Africa, men with secondary education or higher were less likely to engage in HIV risk behaviour. However, it was not the case in Zimbabwe, where bivariate analysis reveal that education level was not associated with condom use during sex with the most recent partner. Age, residence, marital status and wealth status were statistically significant factors influencing condom use. Consistently, in all countries, older men were less likely to use a condom during sex with a recent partner. Men living in urban areas and those who were single had a higher probability of using a condom with a most recent partner. The study also observed that condom use was higher among men who belonged to rich households (Table 1). Our study further reveals that HIV prevalence rates for men aged 15–59 in Southern Africa range between 8.3% in Zambia to 19.6% in Lesotho. These rates are among the highest in sub-Saharan Africa. HIV prevalence rate is lower among men with secondary education or higher, except in Zambia, where the result is the opposite (Supplementary Table 1).

**Table 1:** Percentage distribution of men who used condom with most recent partner by background characteristics, Southern Africa.

	DHS 2014 *N* = 2255	DHS 2011 *N* = 3521	DHS 2013 *N* = 3229	DHS 2016 *N* = 2935	DHS 2015 *N* = 5855	DHS 2018 *N* = 9224
Background characteristics	Lesotho	Mozambique	Namibia	South Africa	Zimbabwe	Zambia
*Age*	***	***	***	***	***	***
15–24	76.3	33.3	79.2	74.1	63.1	41.5
25–34	59.3	16.7	64.6	52.6	23.3	22.8
35–44	42.2	9.4	46.7	38.9	17.1	16.4
45–54	41.8	4.0	30.3	30.9	20.6	12.4
55–59	27.7	1.6	23.0	18.3	–	9.3
*Residence*	***	***	**	*	***	***
Urban	66.8	34.9	61.4	48.7	34.1	28.8
Rural	53.8	8.5	55.7	53.9	27.3	20.7
*Marital status*	***	***	***	***	***	***
Never married	79.2	46.7	80.7	69.4	81.6	50.8
Married	40.9	8.3	30.5	25.2	11.9	12.6
Formerly married	56.8	22.2	61.4	59.0	70.8	42.1
*Age at first sex*	***	**	**	ns	***	**
Below 15 years	66.7	26.8	67.6	55.4	40.3	27.1
15–24 years	57.9	16.9	58.2	49.7	31.6	24.0
25+	41.7	16.4	52.4	45.4	14.1	17.2
*Education level*	***	***	***	***	ns	***
None	35.6	2.0	49.4	31.9	15.3	16.6
Primary	52.3	11.1	51.3	37.2	28.7	16.8
Secondary	70.3	40.8	63.9	54.8	30.9	29.8
Higher	67.5	53.5	57.5	42.2	27.7	29.2
*Literacy*	***	***	***	ns	ns	***
No	36.9	5.1	42.9	42.9	32.0	17.2
Yes	62.8	24.5	60.9	50.7	29.8	25.6
*Wealth status*	***	***	*	*	*	***
Poor	46.1	3.6	55.0	48.5	27.5	20.6
Middle	60.5	12.1	63.6	58.2	29.2	23.6
Rich	65.2	33.5	59.7	47.9	32.3	27.3
*Working status*	ns	***	***	***	***	***
No	59.1	40.5	68.1	59.6	40.0	39.4
Yes	57.8	15.1	54.5	41.9	26.9	21.5
*Circumcision status*	ns	*	ns	ns	***	***
No	59.2	20.2	60.2	45.8	28.5	21.3
Yes	58.0	16.3	56.3	53.3	39.3	30.4
Total	58.3%	18.2%	59.1%	50.2%	29.9%	24.0%

***p < 0.0001; **p < 0.01; *p < 0.05; *ns* not significant.

### Multiple sexual partnerships

The overall patterns in the prevalence of men who had multiple sexual partners were lowest in Namibia (13.7%) and highest in Lesotho (32.9%). The findings show that education was inversely related to having multiple sexual partnerships in men in Lesotho, Namibia, South Africa, and Zambia. An increase in the level of education was associated with a higher chance of having multiple sexual partners. Bivariate analysis shows that a man’s age and age at first sex were significantly associated with having multiple partnerships (*p* < 0.001) in all countries. Findings reveal that as age increases, the risk of engaging in multiple sexual partners reduces among men in the region. Men who began having sexual intercourse at an early age and those from rich households had a higher probability of engaging in multiple partnerships. Men who were living in rural areas from Namibia, South Africa, Zimbabwe and Zambia were engaging in multiple partnerships more than their counterparts in urban areas. In terms of marital status, the never-married men had higher proportions of engaging in multiple sexual relations.

### Uptake of HIV testing

The overall pattern in the prevalence of men who had an HIV test in the last 12 months prior to the DHS was lowest in Mozambique (23%) and highest in Zambia (77.9%). Our findings show that education was positively related to the uptake of an HIV test among men in all the countries. Similarly, in almost all the countries, men who belonged to rich households had a higher proportion of HIV uptake. Living in urban areas was associated with a higher chance of taking an HIV test in all the countries (*p* < 0.000). Bivariate analysis shows that a man’s age and age at first sex were significantly associated with HIV uptake except for South Africa, where an HIV test uptake was not associated with age at first sex. Findings reveal that the uptake of HIV tests was higher among men who were circumcised in countries except for Mozambique. In terms of work status, men who were working had higher proportions of HIV uptake.

### Determinants of HIV transmission risk behaviour among men

Multivariate binary logistic regressions were conducted on a pooled dataset to examine the influence of independent correlates of HIV transmission risk factors among sexually active men aged 15–59 years in Southern Africa. The study found that the age of a man, place of residence, household wealth status and employment status were significantly associated with all the three HIV risk factors examined in this study; that is condom use during sex with the most recent partner, multiple sexual partnerships and HIV testing.

Our study suggests that increasing age was associated with a higher HIV risk behaviour in men. The results show that men in older age groups had lower odds of using condoms during sex with a most recent partner (AOR = 0.63; 95% CI: (0.49–0.81); *p* < 0.001). Men living in rural areas (AOR = 0.72; 95% CI: (0.66–0.78); *p* < 0.001) and those who were married (AOR = 0.11; 95% CI: (0.10–0.12); *p* < 0.001) or formerly married were (AOR = 0.59; 95% CI: (0.50–0.69); *p* < 0.001), respectively, less likely to use a condom during sex with most recent partners than their counterparts in defined reference categories. Men with secondary or higher levels of education had higher odds of using a condom during a sexual encounter with the most recent partner, though the results were not significant. Similarly, men who belonged to middle or high wealth status families were more likely to use a condom during sex with most recent partners (AOR = 1.25; 95% CI: 1.13–1.39; *p* < 0.001) and (AOR = 1.30; 95% CI: 1.19–1.42; *p* < 0.001), respectively. Our study also found that men who were circumcised were more likely to use condoms during sexual intercourse with a recent partner (AOR = 1.17; 95% CI: 1.08–1.27; *p* < 0.001).

The study further reveals that older men had lower odds of engaging in multiple sexual partnerships in the region (*p* < 0.001) compared to young men. Men living in rural areas and those who were formerly married were more likely to have multiple sexual partnerships than their counterparts living in urban areas and the never married, respectively. The study further found that education level was not associated with engaging in multiple sexual partnerships. Study results also show that men who belonged to moderate, and those who belonged to rich households were more likely to have multiple sexual partnerships than those from poor households (AOR = 1.22; 95% CI: 1.10–1.35; *p* < 0.001) and (AOR = 1.36; 95% CI: 1.24–1.50; *p* < 0.001), respectively. Additionally, the study also found that men who were circumcised were more likely to have multiple sexual partnerships (AOR = 1.27; 95% CI: 1.17–1.37; *p* < 0.001).

Regarding HIV testing uptake, our study found that age, residence, marital status, education, household wealth status and employment status were associated with the uptake of HIV testing among sexually active men in the region. The odds of HIV testing were higher among men in the age groups 25–34 and 35–44 years (AOR = 2.04; 95% CI: 1.85–2.25; *p* < 0.001) and (AOR = 2.13; 95% CI: 1.89–2.39; *p* < 0.001), respectively. Men living in rural areas had lower odds (AOR = 0.83; 95% CI: 0.75–0.92) of taking an HIV test compared to their counterparts living in urban areas. Married men and those who were formerly married (AOR = 1.51; 95% CI: 1.38–1.66) and (AOR = 1.36; 95% CI: 1.16–1.59), respectively, were more likely to take an HIV test than those who never-married men. Men with secondary or higher levels of education were more likely to take an HIV test than men with no education. Similarly, men who belonged to rich households were more likely to take an HIV test (AOR = 1.21; 95% 1.10–1.34; *p* < 0.001) compared to those who belonged to a poor household. This study has established that being circumcised or being employed was not associated with the uptake of an HIV test among men (Tables 2–4).

**Table 2:** Percentage distribution of men with multiple sexual partnerships by background characteristics, Southern Africa.

	DHS 2014 *N* = 2255	DHS 2011 *N* = 3521	DHS 2013 *N* = 3229	DHS 2016 *N* = 2935	DHS 2015 *N* = 5855	DHS 2018 *N* = 9224
Background characteristics	Lesotho	Mozambique	Namibia	South Africa	Zimbabwe	Zambia
*Age*	***	***	***	***	***	***
15–24	37.2	33.2	16.9	32.4	25.0	21.2
25–34	41.1	36	16.2	21.4	21.7	22.6
35–44	26.5	31.5	9.8	12.1	17.6	18
45–54	18.6	23.2	8.3	6.6	16.5	16.8
55–59	11.3	20.6	6.7	5.3	–	12.9
*Residence*	*	ns	*	**	**	***
Urban	38.6	33.7	12.3	17.4	22.6	16.3
Rural	29.9	30.5	15.7	23.5	19.2	22.3
*Marital status*	**	*	***	***	***	ns
Never married	37.3	35.3	18.8	26.5	26.9	21.6
Married	29.5	30.2	7.1	9.7	17.9	19.2
Formerly married	31.5	35.2	10.9	22	30.7	19.5
*Age at first sex*	***	***	**	***	***	***
Below 15 years	45.9	45.7	20.3	28.9	32.2	29.2
15–24 years	32.3	29.8	12.8	18	21.8	19.1
25+	12.6	10.2	9.7	5.7	7.1	6.7
*Education level*	**	ns	*	***	ns	*
None	22.1	34.8	8.6	9.1	13.6	16.6
Primary	30.0	28.0	12.4	11.6	19.5	19.9
Secondary	37.7	38.1	14.1	20.7	21.3	20.9
Higher	41.9	32.4	18.1	21.0	18.6	16.2
*Literacy*	***	ns	*	**	ns	ns
No	23.7	30.4	9.6	14.4	17.4	18.5
Yes	34.9	32.3	14.1	19.5	20.7	20.2
*Wealth status*	*	**	ns	**	ns	**
Poor	29.2	27.2	14.2	17.0	18.9	17.3
Middle	29.3	32.4	12.9	26.0	20.0	21.5
Rich	36.9	35.3	13.7	18.0	22.1	21.5
*Working status*	**	**	ns	*	ns	ns
No	28.8	24.2	14.7	21.5	19.7	21.5
Yes	35.2	32.8	13.2	17.1	20.7	19.6
*Circumcision status*	ns	ns	ns	**	ns	ns
No	28.4	30.4	14.2	15.0	20.4	19.7
Yes	34.4	32.9	12.4	22.1	20.9	20.4
Total	32.9%	31.7%	13.7%	19.2%	20.5%	19.9%

***p < 0.0001; **p < 0.01; *p < 0.05; *ns* not significant.

**Table 3:** Percentage distribution of men who tested for HIV and received results in the last 12 months by background characteristics, Southern Africa.

	DHS 2014 *N* = 2255	DHS 2011 *N* = 3521	DHS 2013 *N* = 3229	DHS 2016 *N* = 2935	DHS 2015 *N* = 5855	DHS 2018 *N* = 9224
Background characteristics	Lesotho	Mozambique	Namibia	South Africa	Zimbabwe	Zambia
*Age*	***	***	***	***	***	***
15–24	53.0	17.7	41.2	58	47.5	61.5
25–34	74.2	31.9	78.8	78.5	76.9	89.7
35–44	78.8	24.8	82.2	80.4	78.2	89.9
45–54	77.7	22.5	75.8	77.2	73.6	86.6
55–59	76.7	15.8	74.0	76.7	–	80.7
*Residence*	***	***	***	***	***	***
Urban	77.3	36.3	71.8	74.1	70.1	81.0
Rural	60.8	15.5	54.1	66.2	61.7	75.5
*Marital status*	***	***	***	***	***	***
Never married	55.6	18.1	71.8	64.1	47.1	62.1
Married	78.4	25	54.1	82.3	78.2	90.1
Formerly married	75.2	32.3	67.6	83.6	76.2	83.9
*Age at first sex*	**	**	**	ns	**	***
Below 15 years	61.5	21.2	65.3	75.5	66	75.9
15–24 years	70.9	25.0	71.8	76.5	73.6	85.1
25+	71.2	41.5	81.0	74.7	77.2	84.6
*Education level*	***	***	***	***	***	***
None	62.6	9.5	56.7	69.2	53.7	67.8
Primary	60.2	16.6	55.1	63.0	52.4	70.5
Secondary	71.0	41.1	65.4	71.5	65.8	82.1
Higher	85.0	77.6	85.7	84.2	84.7	92.8
*Literacy*	***	***	***	**	***	***
No	55.1	11.8	49.5	61.2	46.2	68.2
Yes	68.7	28.5	65.7	72.4	65.9	80.1
*Wealth status*	***	***	***	*	ns	ns
Poor	59.5	8.2	54.6	68.9	64.0	76.9
Middle	60.2	14.0	62.1	71.6	64.6	76.7
Rich	73.9	39.8	72.1	74.8	65.3	79.3
*Working status*	**	*	***	***	***	***
No	62.4	19.5	47.3	65.2	53.6	61.8
Yes	69.2	24.0	76.6	79.0	70.5	83.2
*Circumcision status*	***	ns	***	***	***	***
No	54.6	24.9	60.7	65.8	61.5	76.3
Yes	70.9	21.5	73.7	76.4	83.7	81.7
Total	66.4%	23.%	64.0%	71.6%	64.7%	77.9%

***p < 0.0001; **p < 0.01; *p < 0.05; *ns* not significant.

**Table 4:** Results of pooled multivariate analyses examining the effect of individual-level factors on HIV risk behaviours among men in Southern Africa.

	Condom use with most recent partner		Multiple partnerships		HIV testing in last 12 months	
Background characteristics	AOR	95% CI	AOR	95% CI	AOR	95% CI
*Age*						
15–24	1		1		1	
25–34	1.14*	(1.02–1.28)	1.05	(0.94–1.18)	2.04***	(1.85–2.25)
35–44	1.07	(0.94–1.21)	0.75***	(0.66–0.85)	2.13***	(1.89–2.39)
45–54	0.92	(0.80–1.07)	0.63***	(0.54–0.73)	1.80***	(1.58–2.04)
55–59	0.63***	(0.49–0.81)	0.45***	(0.35–0.59)	1.51***	(1.25–1.83)
*Residence*						
Urban	1		1		1	
Rural	0.72***	(0.66–0.78)	1.24***	(1.13–1.36)	0.83***	(0.75–0.92)
*Marital status*						
Never married	1		1		1	
Married	0.11***	(0.10–0.12)	0.89*	(0.80–1.00)	1.51***	(1.38–1.66)
Formerly married	0.59***	(0.50–0.69)	1.25*	(1.05–1.49)	1.36***	(1.16–1.59)
*Age at first sex*						
Below 15 years	1		1		1	
15–24 years	1.04	(0.93–1.16)	0.60***	(0.55–0.66)	1.04	(0.94–1.15)
25+	1.04	(0.85–1.26)	0.22***	(0.17–0.28)	1.02	(0.84–1.22)
*Education level*						
None	1		1		1	
Primary	0.81*	(0.67–0.97)	0.89	(0.74–1.08)	1.44***	(1.24–1.66)
Secondary	1.11	(0.91–1.36)	0.87	(0.71–1.05)	3.10***	(2.64–3.64)
Higher	1.04	(0.82–1.32)	0.83	(0.66–1.05)	5.19***	(4.18–6.45)
*Literacy*						
No	1		1		1	
Yes	1.69***	(1.47–1.94)	1.09	(0.96–1.23)	1.35***	(1.22–1.49)
*Wealth status*						
Poor	1		1		1	
Middle	1.25***	(1.13–1.39)	1.22***	(1.10–1.35)	1.03	(0.93–1.14)
Rich	1.30***	(1.19–1.42)	1.36***	(1.24–1.50)	1.21***	(1.10–1.34)
*Working status*						
No	1		1		1	
Yes	0.73***	(0.66–0.81)	1.22***	(1.12–1.34)	1.00	(0.92–1.09)
*Circumcision status*						
No	1		1		1	
Yes	1.17***	(1.08–1.27)	1.27***	(1.17–1.37)	1.03	(0.95–1.11)

***p < 0.0001; **p < 0.01; *p < 0.05.

### Factors associated with HIV risk behaviour among men in the individual countries

Tables 5 and 6 show country-level multivariate analysis of several independent correlates that are associated with condom use and HIV testing. The findings in Table 5 show that there are country-level variations in the way independent correlates influence condom use during sex with a most recent sexual partner among men in Southern African countries. In the full regression model, education was significantly associated with condom use among men only in Mozambique and Zimbabwe. An increase in the level of education was associated with higher odds of using condoms among men in the two countries. Findings show that an increase in the age of men was associated with lower odds of using condoms in Lesotho, Mozambique, Namibia and South Africa. The results were the opposite in Zimbabwe, where older men were more likely to use a condom during sexual debut with most recent partners compared to young men. In Zambia, age was not significantly associated with condom use. The odds of condom use were lower among men living in rural areas in all the countries except for South Africa. Compared to the poor, men in Lesotho from a middle wealth quantile (AOR = 1.47, 95% CI = 1.07–2.00) and those from both moderate (AOR = 2.79, 95% CI = 1.68–4.68*)* and rich households (AOR = 4.90, 95% CI = 2.93–8.20) in Mozambique were more likely to use condoms during sexual intercourse with most recent partners. Our study also found that the level of education was not significantly associated with having multiple sexual partnerships in all the countries. Age at first sex was found to be inversely associated with having multiple partnerships; men who initiate sexual debut late were less likely to have multiple sexual partnerships (Supplementary file 2: Table 2).

**Table 5:** Results of multivariate analyses examining the effect of individual-level factors on HIV risk behaviour among men by country in Southern Africa countries.

	Condom use with most recent partner					
	Lesotho	Mozambique	Namibia	South Africa	Zimbabwe	Zambia
Background characteristics	AOR (95% CI)	AOR (95% CI)	AOR (95% CI)	AOR (95% CI)	AOR (95% CI)	AOR (95% CI)
*Age*						
15–24	1	1	1	1	1	1
25–34	0.94 (0.66–1.34)	1.07 (0.79–1.45)	0.88 (0.67–1.16)	0.88 (0.67–1.16)	1.06 (0.79–1.42)	1.12 (0.89–1.41)
35–44	0.63* (0.66–1.34)	0.79 (0.52–1.18)	0.65* (0.47–0.89)	0.65* (0.47–0.89)	1.67** (1.25–2.24)	1.13 (0.88–1.44)
45–54	0.70 (0.66–1.34)	0.28*** (0.14–0.53)	0.42*** (0.28–0.62)	0.42*** (0.28–0.63)	2.20*** (1.51–3.20)	0.86 (0.66–1.13)
55–59	0.37** (0.66–1.34)	0.16*** (0.06–0.41)	0.38** (0.21–0.68)	0.24*** (0.14–0.43)	–	0.69 (0.43–0.10)
*Residence*						
Urban	1	1	1	1	1	1
Rural	0.69* (0.50–0.94)	0.53** (0.36–0.76)	0.69** (0.55–0.87)	0.97 (0.77–1.23)	0.75** (0.62–0.90)	0.84* (0.73–0.97)
*Marital status*						
Never married	1	1	1	1	1	1
Married	0.22*** (0.16–0.31)	0.21*** (0.15–0.29)	0.14*** (0.10–0.18)	0.23*** (0.17–0.30)	0.02*** (0.02–0.03)	0.15*** (0.12–0.18)
Formerly married	0.48** (0.30–0.79)	0.59** (0.37–0.93)	0.53* (0.33–0.85)	1.00 (0.62–1.60)	0.43*** (0.30–0.61)	0.75 (0.55–1.02)
*Age at first sex*						
Below 15 years	1	1	1	1	1	1
15–24 years	1.15 (0.83–1.58)	0.75 (0.55–1.02)	0.96 (0.71–1.31)	1.08 (0.78–1.49)	1.14 (0.80–1.62)	1.05 (0.87–1.25)
25+ years	0.48 (0.63–1.85)	1.88 (0.39–0.10)	1.93* (1.08–3.44)	1.94* (1.03–3.65)	0.77 (0.49–1.23)	0.94* (0.67–1.31)
*Education level*						
No education	1	1	1	1	1	1
Primary	0.74 (0.45–1.23)	2.94* (1.28–6.75)	0.71 (0.46–1.08)	0.77 (0.42–1.43)	2.58* (1.08–6.16)	0.79 (0.55–1.12)
Secondary	1.01 (0.59–1.74)	5.62*** (2.28–3.89)	0.83 (0.53–1.30)	1.05 (0.56–2.00)	2.41* (1.01–5.75)	1.23 (0.84–1.81)
Tertiary	0.95 (0.47–1.89)	10.23*** (3.89–16.89)	0.79 (0.45–1.37)	0.74 (0.37–1.50)	2.27 (0.93–5.54)	1.31 (0.84–2.05)
*Literacy*						
Illiterate	1	1	1	1	1	1
Literacy	2.10** (1.07–3.27)	1.34 (0.85–2.10)	1.97** (1.27–3.06)	0.95 (0.62–1.45)	1.29 (0.86–1.94)	1.21** (0.97–1.49)
*Wealth status*						
Poor	1	1	1	1	1	1
Moderate	1.47* (1.07–2.00)	2.79*** (1.68–4.63)	1.20 (0.90–1.60)	1.42 (1.03–1.98)	0.86 (0.68–1.09)	1.06 (0.90–1.25)
Rich	1.29 (0.94–1.77)	4.90*** (2.93–8.20)	0.99 (0.74–1.33)	1.25 (0.96–1.63)	0.93 (0.74–1.15)	1.13 (0.97–1.31)
*Working status*						
No	1	1	1	1	1	1
Yes	1.07 (0.83–1.39)	0.75 (0.53–1.04)	0.84 (0.67–1.06)	0.89 (0.69–1.15)	0.89 (0.72–1.09)	0.92 (0.74–1.14)
*Circumcision status*						
No	1	1	1	1	1	1
Yes	1.09 (0.83–1.42)	0.74* (0.58–0.94)	1.06 (0.84–1.34)	1.17 (0.92–1.50)	0.92 (0.69–1.22)	1.10 (0.95–1.27)

***p < 0.0001; **p < 0.01; *p < 0.05.

**Table 6:** Results of multivariate analyses examining the effect of individual-level factors on HIV risk behaviour among men by country in Southern Africa countries.

	HIV testing in the last 12 months prior to survey					
	Lesotho	Mozambique	Namibia	South Africa	Zimbabwe	Zambia
Background Characteristics	AOR (95% CI)	AOR (95% CI)	AOR (95% CI)	AOR (95% CI)	AOR (95% CI)	AOR (95% CI)
*Age*						
15–24	1	1	1	1	1	1
25–34	2.15 (1.60–2.90)***	1.67 (1.22–2.30)**	3.11 (2.35–4.12)***	1.56 (1.15–2.11)*	1.41 (1.14–1.74)**	1.83 (1.50–2.23)***
35–44	2.96 (1.89–4.62)***	1.23 (0.87–1.74)	4.04 (3.02–5.40)***	1.61 (1.13–2.31)*	1.18 (0.92–1.52)	1.57 (1.23–2.02)***
45–54	3.02 (1.87–4.89)***	1.01 (0.70–1.45)	2.68 (1.85–3.88)***	1.29 (0.84–1.99)	0.90 (0.68–1.20)	1.23 (0.95–1.59)
55–59	3.34 (1.83–6.11)***	0.77 (0.45–1.32)***	2.72 (1.59–4.68)***	1.25 (0.74–2.11)	_	0.74 (0.50–1.08)
*Residence*						
Urban	1	1	1	1	1	1
Rural	0.56 (0.42–0.77)***	0.97 (0.72–1.30)	0.70 (0.56–0.88)	0.85 (0.67–1.09)	1.00 (0.86–1.18)	0.81 (0.65–1.01)
*Marital status*						
Never married	1	1	1	1	1	1
Married	1.80 (1.33–2.44)***	2.22 (1.61–3.07)***	1.23 (10.97–1.56)	1.73 (1.29–2.31)***	2.45 (2.02–2.97)***	3.70 (2.94–4.66)***
Formerly married	1.37 (0.84–2.24)	2.66 (1.67–4.00)***	0.62 (0.38–0.99)*	1.99 (1.13–3.51)*	2.24 (1.62–3.10)***	1.97 (1.39–2.80)***
*Age at first sex*						
Below 15 years	1	1	1	1	1	1
15–24 years	0.93 (0.68–1.27)	1.14 (0.87–1.49)	1.07 (0.80–1.42)	0.92 (0.68–1.24)	1.19 (0.91–1.56)	1.18 (0.98–1.42)
25+ years	0.72 (0.42–1.25)	2.59 (0.98–6.81)	1.32 (0.76–2.29)	0.86 (0.42–1.74)	1.09 (0.77–1.53)	0.70 (0.48–1.02)
*Education level*						
No education	1	1	1	1	1	1
Primary	0.93 (0.60–1.43)	1.59 (1.04–2.42)*	1.10 (0.78–1.55)	0.77 (0.46–1.31)	0.78 (0.42–1.46)	1.09 (0.79–1.50)
Secondary	1.64 (0.99–2.71)	4.41 (2.68–7.26)***	1.65 (1.14–2.40)*	1.09 (0.60–1.98)	1.31 (0.70–2.46)	1.93 (1.37–2.72)***
Tertiary	1.99 (1.01–3.92)*	15.52 (7.61–31.67)***	2.35 (1.30–4.25)*	1.42 (0.69–2.91)	2.82 (1.45–5.50)**	2.48 (1.48–4.15)**
*Literacy*						
Illiterate	1	1	1	1	1	1
Literacy	1.99 (1.42–2.79)***	1.99 (1.42–2.79)***	1.79 (1.32–2.43)***	1.51 (0.99–2.30)	1.30 (0.99–1.72)	1.43 (1.22–1.68)***
*Wealth status*						
Poor	1	1	1	1	1	1
Moderate	0.86 (0.65–1.15)	1.73 (1.19–2.53)*	0.98 (0.78–1.23)	1.18 (0.85–1.66)	1.01 (0.83–1.22)	1.03 (0.85–1.24)
Rich	1.15 (0.85–1.54)	5.13 (3.64–7.25)***	1.25 (0.94–1.67)	1.43 (1.09–1.88)*	1.10 (0.93–1.29)	1.33 (1.11–1.58)**
*Working status*						
No	1	1	1	1	1	1
Yes	0.85 (0.67–1.08)	0.98 (0.71–1.36)	1.61 (1.30–2.00)***	1.02 (0.80–1.29)	1.03 (0.88–1.22)	1.11 (0.96–1.30)
*Circumcision status*						
No	1	1	1	1	1	1
Yes	2.05 (1.61–2.61)***	0.54 (0.42–0.70)***	1.48 (1.18–1.84)**	1.60 (1.28–2.00)***	3.12 (2.25–4.34)***	1.40 (1.20–1.63)***

***p < 0.0001; **p < 0.01; *p < 0.05

Findings in Table 6 show that uptake of HIV testing among sexually-active men varies across countries in Southern Africa. Multivariate regression analysis shows that education was significantly associated with HIV testing among men in Lesotho, Mozambique, Namibia, Zimbabwe and Zambia. An increase in the level of education was associated with higher odds of HIV testing uptake among men in these countries. Results further show that in Lesotho and Namibia, older men were more likely to take an HIV test compared to young men aged 15–24. In Mozambique and Zambia, men aged 55–59 were less likely to take an HIV test compared to men aged 15–24. The odds of HIV testing uptake were lower among men living in rural areas only in Lesotho. Compared to men from poor households, those from both moderate (AOR = 1.73, 95% CI = 1.19–2.53) and rich households (AOR = 5.13, 95% CI = 3.64–7.25) in Mozambique, were more likely to take an HIV test. Furthermore, men from rich households (AOR = 1.43, 95% CI = 1.09–1.88) in South Africa, were more likely to take an HIV test. The study found that age at first sex was not associated with undertaking an HIV test among sexually active men in all the countries. Additionally, in all the countries except for Mozambique, circumcised men were more likely to undertake an HIV test.

## Discussion

This study sought to analyse the influence of education and other individual correlates on HIV transmission risk behaviour in men, and examine differentials of the risk factors across countries in Southern Africa. The study utilised the most recent cross-sectional data collected in the respective countries. The study focused on the influence of individual-level factors on HIV risk behaviour among men in the Southern African region, and also at the country level. Our review of the literature reveals that there is little or no known evidence of a comprehensive study of this nature that has been conducted to inform HIV risk behaviour among sexually active men in the region. Thus, analysis done using pooled data bolsters the importance of the findings to inform regional HIV and AIDS public health policy and programmes to further reduce the regional HIV prevalence.

Even though the use of condoms is one of the most effective ways to reduce the sexual transmission of HIV, the rate at which condoms are used in countries in the region is still unacceptably low. The study findings show that overall, condom use during sex with most recent partners is not influenced by the level of education among men. Prior studies have identified multiple sexual relations as a major hindrance to reducing HIV transmission in SSA (Deeks et al., 2016; Mutinta, 2014; Nalukwago et al., 2018; Onoya et al., 2015). Peer influence, parental and societal indifference to polygamy and male dominance in sexual relation decisions as major factors influencing men’s decision to engage in multiple sexual relations. Our study found that education had no influence on men’s decision to engage in multiple sexual partnerships. However, education level showed a positive association with the uptake of HIV testing among men. The study also shows that there are major disparities in the prevalence of HIV testing among men in different educational sub-categories across countries. Furthermore, we found that married men and those who were formerly married were more likely to use a condom and take an HIV test than never-married men. This could imply that this positive sexual behaviour being adopted by married men is meant to protect their spouses within the marriage institution. The findings of this study have implications for both shaping public health policy and re-designing sexual reproductive health programmes in the region, considering the COVID-19 pandemic that may pose significant challenges in accessing sexual reproductive health services, especially among marginalised communities.

Recent studies on education and HIV/AIDS in SSA have shown sound evidence of the association between education and reduced risk of HIV transmission, suggesting the need for countries to invest in the education sector in order to maximise social behaviour change in men (De Neve et al., 2015; Leon et al., 2017; Pettifor et al., 2008). However, it is important to note that because of the socio-cultural heterogeneity of sub-Saharan African countries, the influence of education on HIV risk behaviour among men may not be similar in all the countries. Our study findings further reveal that, at the regional level, an increasing level of education was not associated with a higher probability of using condoms during a sexual encounter with most recent partners among men. This means that in the pooled analysis, the educational level of a man did not play a significant role in determining a positive decision to avoid HIV transmission risk. Individual country-level analysis shows that an increasing level of education was associated with increased odds of using condoms among men in Mozambique and Zimbabwe. This, therefore, shows a positive association between education and condom use in these two countries, implying that promoting education should be a priority in influencing safer sex behaviour among men in Mozambique and Zimbabwe.

Other studies have also reported that men who are well educated are better able to seek more accurate information with which to assess their risks and develop preventative measures (Exavery et al., 2012; Jung et al., 2013; Leon et al., 2017; Manlove et al., 2008; Zhao et al., 2012). This, therefore, suggests that men with low levels of education may be at high risk of contracting the HIV virus. Related studies confirm this finding in other SSA countries. A study conducted in 2017 in four countries (Ghana, Kenya, Tanzania and Cameroon) found that people with no or primary education had a greater risk of acquiring HIV than those with secondary education or higher (Leon et al., 2017). Theoretically, multiple sexual partnerships can play a negative role in the spread of HIV in most societies through multiple sexual networks (Lurie and Rosenthal, 2010). Though no conclusive evidence is available on its relationship with HIV prevalence, multiple sexual partnerships might increase the risk of acquiring HIV if coupled with inconsistent condom use or non-safe sex practices (Maher et al., 2011; SAfAIDS, 2015). Some recent studies have reported multiple sexual partnerships and inconsistent condom use as among the key drivers of HIV transmission among men in sub-Saharan Africa (Manjengwa et al., 2019). However, this study found that educational level was not associated with men’s decision to engage in multiple sexual partnerships in the region. There were no differences in proportions of men engaging in multiple sexual relations by education level. The decision to engage in multiple sexual relations appears to be a personal intrinsic choice to satisfy one’s sexual desire rather than a decision informed by education. This finding has implications for the success of behaviour change campaigns, which aim at encouraging men to stick to one faithful partner. A significant component of social behaviour change campaigns in SSA has a target to reduce multiple partnerships in order to reduce exposure to HIV infection (Khidir et al., 2020; SAfAIDS, 2015).

HIV testing is regarded as one important step in the prevention of HIV transmission, especially among men with more than a sexual partner (Awopegba et al., 2021; Olakunde et al., 2020a). It is expected that men who know their HIV status should make an informed decision about prevention measures; that is correct and consistent use of condoms to avoid spreading the HIV virus or preventing infection of their partners, if HIV positive. Studies conducted in South Africa, Nigeria and Ghana on HIV testing coverage found that men with a higher level of education were more likely to test for HIV (Awopegba et al., 2021; Nyarko and Sparks, 2020; Olakunde et al., 2020a). These studies are consistent with the findings of our study. Other studies have shown that educated persons are highly likely to be knowledgeable about disease preventive measures and be able to make informed decisions to present themselves early for HIV treatment (Hachfeld et al., 2019; Mulemena et al., 2020). Non-acceptance of HIV testing among men with lower levels of education has an implication for the early initiation of HIV-positive clients on antiretroviral treatment (ART). Community and school-based HIV prevention campaigns championing HIV self-testing will be key to reaching universal HIV testing coverage.

Community and school-based interventions supporting self-HIV testing campaigns have been documented to be among the successful approaches to enhancing acceptance and uptake of HIV testing in Tanzania, Uganda, Malawi and Kenya (Chamie et al., 2014; Harper et al., 2018; Kumwenda et al., 2019; Kurth et al., 2015; Mangenah et al., 2019; Njau et al., 2021). Njau et al. (2021) in Tanzania found that HIV self-testing initiatives were workable to implement in settings where the majority of community members had positive attitudes and supportive perceived norms towards HIV prevention. In South Africa, Mokgatle (2017) proposed extending school-based self-HIV testing interventions to communities because of its effectiveness in promoting acceptance of HIV testing. In Kenya, Harper et al. (2018) reported that school-based HIV testing campaigns had the potential to become institutionalised in school settings in order to maintain their long-term sustainability (Harper et al., 2018; Mokgatle and Madiba, 2017; Njau et al., 2021).

The reviewed literature and analysis of this study show that education plays a part in HIV transmission among men in some countries in Southern Africa. However, the influence of education on various HIV risk factors is not uniform. Despite similarities in socio-economic and cultural contexts among countries in the region, analyses in this study present heterogeneity in the influence of education on different HIV risk factors. Other studies on SSA have found that other factors (i.e. age, residence, household wealth status, religious affiliation and employment status) were equally important factors influencing HIV transmission in SSA (Asaolu et al., 2016b; Dake et al., 2012; Devine-Wright et al., 2015; Nyarko and Sparks, 2020; Oster, 2012; Voisin et al., 2012). Detailed decomposition analysis of factors’ contribution to the reduction in HIV prevalence at the country level would be useful to inform adequate conclusions on key factors influencing HIV risk behaviour and transmissions in Southern African countries.

## Conclusion

The study showed that education matters in explaining the uptake of HIV testing among sexually active men in Southern African countries. Country-level variations exist in terms of how education influences HIV transmission risk behaviour, such as condom use and HIV testing, among men. Age of men, place of residence, marital status, wealth status and circumcision status were found to be significantly associated with HIV risk behaviour in men. Therefore, investing in education could be one of the potential long-term cost-effective interventions to reduce HIV infections among men in some countries in the region, as it has shown potential to improve HIV testing uptake in men. The study has also established that HIV prevention campaigns targeting men at the country level are yielding different results in the region. Therefore, there is the need to re-evaluate country-level HIV prevention programmes across the region to identify best practices. There is also a need for countries in the sub-region to consider strengthening community social behaviour change communication (SBCC) programmes, aimed at discouraging cultural practices that predispose men to engage in multiple sexual partnerships. Furthermore, empowering young men with comprehensive sexual education through the integration of sexuality education into primary and secondary school curricula could yield long-term benefits in reducing HIV risk behaviour among men. There is a need for qualitative research to explore socio-cultural factors that could explain why educated men engage in risky behaviour, such as multiple sexual partnerships.

### Study limitations

There are several limitations to the DHS studies. First, because the data is cross-sectional, therefore, the researchers could not do causality analyses, which limits the ability to understand the complexities of men’s experiences regarding the risk of HIV transmission behaviours through their life cycle. As a result, the findings highlight the need for additional research, particularly qualitative and longitudinal research, to further the understanding of the complex interplay between the various individual and community factors that shape men’s HIV risk behaviours. However, the male datasets in the DHS programme are comprehensive enough to understand men’s sexual behaviour in detail. Data for other countries in the region was not available on the DHS programme website.

## Supplementary information

Supplementary tables

## Data Availability

Data used in this study are publicly available upon request from the DHS programme website: https://dhsprogram.com/.
