# Supplementary material for: Examining differentials in HIV transmission risk behaviour and its associated factors among men in Southern African countries
Source: Humanit Soc Sci Commun. 2022 Aug 27;9(1):295. doi: 10.1057/s41599-022-01312-3 (PMC9419142; doi:10.1057/s41599-022-01312-3)
Supplement: Supplementary file 1 — Supplementary tables [file 41599_2022_1312_MOESM1_ESM.docx]

**Supplementary file 1: Table 1: HIV prevalence rate among men aged 15-59 years by education level, DHS, Southern Africa**

| **Country** | **Survey** | **No education or primary** | **Secondary or higher** | **Total** |
| --- | --- | --- | --- | --- |
| Lesotho | 2014 DHS | 21.0 (CI: 18.3 – 23.8) | 15.9 (CI: 12.6 – 19.3) | **19.6 (CI: 17.5 – 21.6)** |
| Mozambique | 2015 AIS | 10.6 (CI: 8.8 – 12.3) | 9.2 (CI: 7.5 – 11.0) | **10.2 (CI: 8.9 – 11.6)** |
| Namibia | 2013 DHS | 14.4 (CI: 12.0 – 16.9) | 9.2 (CI: 7.2 – 11.2) | **11.4 (CI: 9.8 – 13.1)** |
| South Africa | 2016 DHS | 15.3 (CI: 9.7 – 20.9) | 14.2 (CI: 11.0 – 17.4) | **13.3 (CI: 11.0 – 15.6)** |
| Zambia | 2018 DHS | 6.8 (CI: 5.7 – 7.9) | 8.0 (CI: 7.1 – 8.9) | **8.3 (CI: 7.5 – 9.1)** |
| Zimbabwe | 2015 DHS | 11.6 (CI: 9.6 – 13.7) | 10.2 (CI: 9.1 – 11.2) | **11.3 (CI: 10.3 – 12.3)** |

| **Supplementary file 2: Table 2: Results of multivariate analyses examining the effect of individual level factors on HIV risk behaviour among men by country in Southern Africa countries** | | | | | | |
| --- | --- | --- | --- | --- | --- | --- |
|  | **Having multiple partnerships** | | | | | |
|  | **Lesotho** | **Mozambique** | **Namibia** | **South Africa** | **Zimbabwe** | **Zambia** |
| **Background**  **Characteristics** | AOR (95% CI) | AOR (95% CI) | AOR (95% CI) | AOR (95% CI) | AOR (95% CI) | AOR (95% CI) |
| **Age** |  |  |  |  |  |  |
| 15-24 | 1 | 1 | 1 | 1 | 1 | 1 |
| 25-34 | 1.26(0.85-1.86) | 1.18(0.93-1.51) | 1.28(0.91-1.80) | 0.66(0.48-0.90)* | 1.09(0.86-1.38) | 1.29(1.03-1.62)* |
| 35-44 | 0.67(0.47-0.95)* | 1.02(0.77-1.36) | 0.91(0.62-1.36) | 0.40(0.25-0.66)*** | 0.94(0.71-1.25) | 0.99(0.78-1.27) |
| 45-54 | 0.50(0.29-0.85)* | 0.69(0.49-0.98)* | 1.06(0.57-1.97) | 0.23(0.13-0.40)*** | 0.87(0.63-1.21) | 0.91(0.70-1.20) |
| 55-59 | 0.29(0.14-0.58)** | 0.60(0.34-1.09) | 0.96(0.35-2.60) | 0.20(0.09-0.44)*** | _ | 0.64(0.44-0.94)* |
| **Residence** |  |  |  |  |  |  |
| Urban | 1 | 1 | 1 | 1 | 1 | 1 |
| Rural | 0.80(0.55-1.17) | 1.17(0.93-1.46) | 1.50(1.08-2.09)* | 1.39(0.35-0.82) | 0.75(0.62-0.90)** | 1.46(1.23-1.73) |
| **Marital status** |  |  |  |  |  |  |
| Never married | 1 | 1 | 1 | 1 | 1 | 1 |
| Married | 1.03(0.74-1.42) | 0.84(0.63-1.12) | 0.34(0.23-0.49)*** | 0.53(0.35-0.82)* | 0.72(0.55-0.94)* | 0.98(0.78-1.23) |
| Formerly married | 1.24(0.81-1.88) | 1.01(0.66-1.55) | 0.53(0.22-1.27) | 1.52(0.88-2.63) | 1.36(0.95-1.95) | 0.97(0.67-1.40) |
| **Age at first sex** |  |  |  |  |  |  |
| Below 15 years | 1 | 1 | 1 | 1 | 1 | 1 |
| 15-24 years | 0.60(0.46-0.80)*** | 0.53(0.42-0.66)*** | 0.65(0.45-0.94)* | 0.62(0.46-0.83)** | 0.59(0.45-0.77)*** | 0.59(0.51-0.69)*** |
| 25+ years | 0.23(0.11-0.48)*** | 0.16(0.06-0.44)*** | 0.69(0.30-1.60) | 0.31(0.11-0.85)* | 0.18(0.11-0.28)*** | 0.19(0.12-0.31)*** |
| **Education level** |  |  |  |  |  |  |
| No education | 1 | 1 | 1 | 1 | 1 | 1 |
| Primary | 1.07(0.65-1.76) | 0.65(0.45-0.66)* | 1.29(0.64-2.62) | 0.82(0.35-1.95) | 1.12(0.33-3.86) | 1.20(0.84-1.71) |
| Secondary | 1.39.(0.81-2.39) | 0.87(0.56-1.34) | 1.53(0.73-3.18) | 1.09(0.46-2.62) | 1.08(0.32-3.65) | 1.29(0.90-1.85) |
| Tertiary | 1.48(0.71-3.11) | 0.69(0.39-1.24) | 2.31(1.03-5.19) | 1.48(0.58-3.77) | 0.90(0.26-3.10) | 1.05(0.69-1.61) |
| **Literacy** |  |  |  |  |  |  |
| Illiterate | 1 | 1 | 1 | 1 | 1 | 1 |
| Literate | 1.21(0.81-1.83) | 1.06(0.82-1.36) | 1.16(0.62-2.16) | 0.95(0.54-1.66) | 1.24(0.89-1.76) | 1.13(0.93-1.38) |
| **Wealth status** |  |  |  |  |  |  |
| Poor | 1 | 1 | 1 | 1 | 1 | 1 |
| Moderate | 0.84(0.60-1.17) | 1.29(0.99-1.67) | 0.87(0.57-1.32) | 1.60(1.14-2.24)* | 1.11(0.89-1.37) | 1.29(1.09-1.54)** |
| Rich | 0.96(0.67-1.37) | 1.54(0.18-2.52)** | 0.97(0.66-1.42) | 1.08(0.79-1.47) | 1.28(1.05-1.55) | 1.35(1.14-1.60)** |
| **Working status** |  |  |  |  |  |  |
| No | 1 | 1 | 1 | 1 | 1 | 1 |
| Yes | 1.35(1.06-1.70)* | 1.90(1.44-2.52)*** | 1.18(0.87-1.60) | 1.23(0.91-1.66) | 1.12(0.92-1.35) | 0.98(0.80-1.20) |
| **Circumcision status** |  |  |  |  |  |  |
| No | 1 | 1 | 1 | 1 | 1 | 1 |
| Yes | 1.39(1.03-1.88)* | 1.09(0.91-1.31) | 0.92(0.68-1.23) | 1.38(1.06-1.18)* | 0.90(0.72-1.14) | 1.07(0.94-1.22) |

*** = p<0.0001; ** = p<0.01; *=p<0.05
